# Pericardial Effusion due to Infliximab Therapy for Ulcerative Colitis

**DOI:** 10.1155/2018/4324592

**Published:** 2018-11-06

**Authors:** Muhammad Mirza, Maira Mirza, Vagishwari Murugesan, Arrel Olano

**Affiliations:** ^1^Department of Internal Medicine, Medstar Washington Hospital Center, USA; ^2^Department of Internal Medicine, Cambridge Health Alliance, USA

## Abstract

Ulcerative colitis is characterized by ulcers and inflammation of the inner lining of the gastrointestinal tract. Antitumor necrosis factor alpha (anti-TNF alpha) agents such as infliximab are drugs that have been used for the treatment of ulcerative colitis for decades. Infliximab is known to be associated with various adverse effects including anti-TNF alpha induced lupus (ATIL). We present a rare case of a 51-year-old female with pericardial effusion secondary to infliximab therapy for treatment of her ulcerative colitis. Discontinuation of infliximab led to resolution of the pericardial effusion.

## 1. Introduction

Anti-TNF alpha therapy has proven its role in the treatment of many immunologic and rheumatologic disorders, but it is not without side effects. It may cause an autoimmune response producing symptoms similar to those of Systemic Lupus Erythematosus (SLE) [1]. Infliximab is responsible for most of these cases (incidence of 0.19%-0.22%) followed by etanercept (incidence of 0.18%) and adalimumab (incidence of 0.10%) [1].

Symptoms of drug induced lupus can range from mild cutaneous lesions to complicated pleural or pericardial effusions and life-threatening pneumonitis or neuritis, to name a few. Laboratory data in these patients is often positive for autoantibodies, including antinuclear antibodies (ANA) and antidouble stranded (ds) DNA antibodies. Diagnosis can be considered if there is a temporal association between the anti-TNF alpha therapy and the symptoms and at least one serologic and one nonserologic American College of Rheumatology (ACR) criterion [1].

The main approach in the treatment of ATIL is withdrawal of the offending agent. At times, steroids and immunosuppressive agents may be required to achieve full resolution of the symptoms. [2]

## 2. Case Report

**Figure 1:**
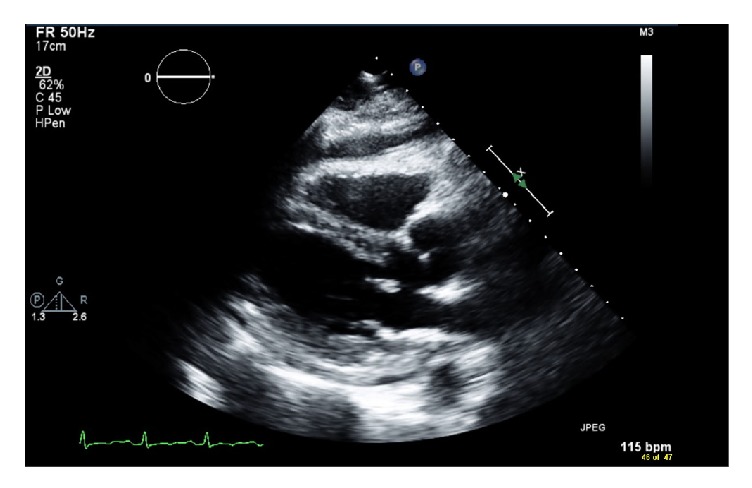
Parasternal long view of the pericardial effusion on transthoracic echocardiogram.

**Figure 2:**
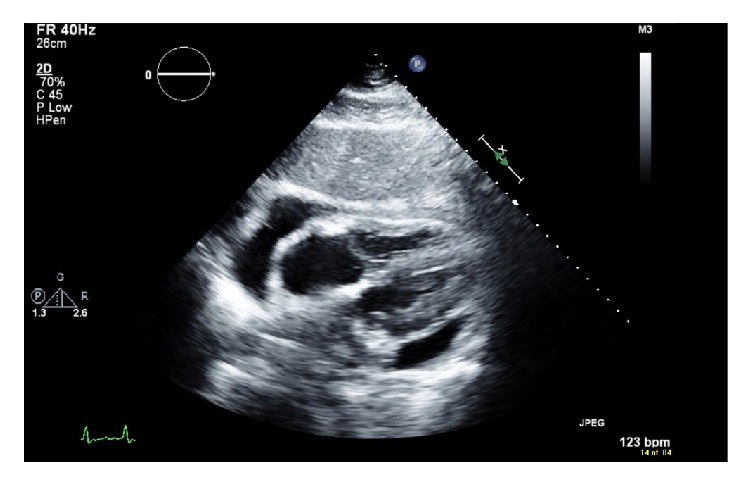
Subcostal view of the pericardial effusion on transthoracic echocardiogram.

We present a case of a 51 year-old white female with a 5-year history of ulcerative colitis. She has been taking mesalamine 1.2 gram (2 tablets two times a day) for 1 year with no complications that were reported. No history of allergies and no history of smoking or alcohol abuse were present. Past medical history was only remarkable for ulcerative colitis and there was no significant past surgical or family history. Around March 2018, she started having increased watery diarrhea with occasional blood (10-12 bowel movements per day from a baseline of 1-2 bowel movements per day) as well as cramping abdominal pain. She went to see her gastroenterologist in clinic. On physical examination, she had diffuse tenderness to palpation of her abdomen. Stool studies including stool cultures, stool ova, and parasites were sent which were negative. ESR and CRP levels were elevated. Therefore, she was thought to be in a moderate to severe ulcerative colitis flare based on the current criteria and was prescribed budesonide multimatrix (MMX) 9 mg once daily. Her abdominal pain improved but the diarrhea persisted. She then received a course of oral prednisone 40 mg daily for one month without any improvement of her symptoms and was subsequently started on infliximab therapy. Prior to initiating infliximab therapy, an interferon gamma release assay, hepatitis panel, varicella zoster antibody, and HIV tests were done which were negative. On 4/13/2018, she received her first dose of infliximab 500 mg based on her weight of 100 kg (5mg/kg). Her symptoms got better during the first week after the infusion; however during her second week, she complained of nonradiating chest pain located at the midsternal region, shortness of breath, and worsening fatigue. She went to a hospital where she was admitted. Her initial vitals were significant for low to normal blood pressure and a persistent tachycardia of up to 110. EKG was negative for any acute changes and a CT-PE was also negative for pulmonary embolism but showed a moderate size pericardial effusion. She was given fluids with no change in the blood pressure, and she continued to remain hypotensive and tachycardic and was eventually transferred to another hospital for concerns of a cardiac tamponade. At the other hospital, a transthoracic echocardiogram was done that showed an ejection fraction of 65-70% and a small to moderate size pericardial effusion that was present more anteriorly and less prominently on the apical, inferior, and subcostal views (Figures 1 and 2). There were no echocardiographic criteria for cardiac tamponade. Based on the difficult anatomical location of the effusion, decision was made to medically manage the patient.

She underwent extensive workup to evaluate the etiology of her pericardial effusion. Viral causes including HIV, monospot test were negative. T-spot was also negative. Due to concerns for a drug-induced lupus from infliximab, ANA and ds-DNA were checked, which were negative. Antihistone Abs were 1.9 (positive). ESR was 70 and CRP was more than 190. There were no signs of serositis, oral ulcers, photosensitivity, blood disorders (leukopenia, anemia, and thrombocytopenia), neurologic disorder, or rash (malar or discoid).

The clinical presentation was not compatible with any other pathology and based on the specified time frame of the presentation, a diagnosis of infliximab induced lupus was made and patient was taken off infliximab therapy.

Her infectious workup for diarrhea including stool culture, stool ova and parasite, and clostridium difficile were negative. A procalcitonin level was also negative. She was in a moderate to severe ulcerative colitis flare and was therefore started on IV methylprednisolone 60 mg daily for 3 days and then transitioned to PO prednisone 40 mg daily. Her shortness of breath and fatigue got better and, after discharge, her diarrhea frequency went back to baseline. After she completed her prednisone taper, she was planned for vedolizumab (antagonist to *α*4*β*7 integrin) therapy for her ulcerative colitis. Vedolizumab is not shown to be associated with drug induced lupus [3] and that is why it was chosen for our patient. She got induction therapy with IV vedolizumab 300 mg at weeks 0, 2, and 6 and was then continued on maintenance therapy with IV vedolizumab 300 mg every 8 weeks. She did not receive infliximab therapy in the future. Post discharge, serum anti-TNF alpha antibodies were checked which were negative.

## 3. Discussion

A specific diagnostic criterion for ATIL has not yet been established yet. However for early diagnosis, a temporal association between symptoms and anti-TNF alpha therapy, at least one serologic ACR criterion (ANA, anti-dsDNA antibodies, and anti-histone antibodies) and one nonserologic ACR criterion (arthritis, serositis, and rash), is needed. Our patient fulfilled all three criteria and was therefore diagnosed with infliximab induced lupus.

The clinical and serological characteristics of ATIL differ from drug induced lupus secondary to other drugs such as hydralazine or procainamide. The clinical presentation consists mostly of arthralgias and cutaneous manifestations. Renal and neurological involvement are far less common. Serositis including pericardial effusions is also rare. ANA is positive in 79% and anti-dsDNA antibodies are positive in 72% of the cases of ATIL [4]. These antibodies are less common in drug-induced-lupus due to hydralazine or procainamide. Antihistone antibodies have been shown to be positive in approximately 95% of patients with drug induced lupus due to hydralazine or procainamide but only 17-57% positive in patients with ATIL [4].

Our patient had negative ANA, anti-dsDNA antibodies but positive anti-histone antibodies which is rare for infliximab induced lupus. Our patient was not on any other medications that could have caused classical drug induced lupus. There has been a rare case of a 59 year old Vietnamese male who was treated with infliximab due to Crohn's disease and he later developed pericarditis. ANA, anti-dsDNA antibodies, anti- histone antibodies were negative but due to the timing of the symptoms and the infliximab therapy, ATIL was considered the likely etiology in this patient and infliximab was discontinued. [5].

Our patient was only given one dose of infliximab 500 mg and her symptoms developed fairly rapidly within 2 weeks which is also very rare to see in patients diagnosed with ATIL, who typically develop symptoms over a longer period of time and with higher doses of anti-TNF alpha drugs. This means that ATIL can present even with lower doses of these drugs and in shorter time durations.

The patient initially did not respond to a course of oral steroids. However, when she was hospitalized and taken off infliximab, she was started on intravenous steroids which helped to calm her symptoms and was then tapered to oral steroids to which she eventually responded and got better.

As previously mentioned, ATIL does present as polyarthralgias in most individuals. [4] There has been a case of a 48 year old female who developed symmetrical polyarthralgias after undergoing treatment with infliximab for ulcerative proctitis. Her serology showed a positive ANA, weakly positive anti-dsDNA antibodies, and positive antihistone antibodies. After discontinuation of the infliximab, her symptoms resolved and repeated autoantibodies were all negative [4].

The pathogenesis of ATIL has not been clearly identified. However, several mechanisms have been proposed. One such theory suggests that anti-TNF *α* decreases the production of Th1 cytokines, therefore driving the immune response towards Th2 cytokine production. This leads to the production of autoantibodies [1]. Our patient did not have any antibodies against infliximab which is also rare to see in patients with infliximab induced lupus. Another hypothesis is the inhibition of TNF-*α* interfering with apoptosis and decreasing CD44 expression, which leads to decreased clearance of nuclear debris and promotes autoantibody production against nuclear antigens [1].

The treatment of ATIL is based upon the cessation of the offending agent. In severe cases, steroids and immunosuppressive agents have also shown to be effective [2]. Alternate therapy is then chosen for the underlying immunological or rheumatological disorder, which in our patient was Ulcerative Colitis. There has been a case of cardiac tamponade caused by infliximab treatment for Crohn's disease in a 30-year-old female. After getting stabilized, she was eventually treated with vedolizumab (antagonist to *α*4*β*7 integrin) therapy for Crohn's disease and had no recurrence of her symptoms [6]. Therefore, our patient was chosen to get vedolizumab therapy for ulcerative colitis which does not act on the TNF alpha pathway and has not been shown to cause drug induced lupus. [3]

Further research is warranted for more definitive criteria for the diagnosis of ATIL, given its complicated course and life threatening manifestations.
